# The *Chlamydia* outer membrane protein OmcB is required for adhesion and exhibits biovar-specific differences in glycosaminoglycan binding

**DOI:** 10.1111/j.1365-2958.2007.06050.x

**Published:** 2007-12-11

**Authors:** Katja Moelleken, Johannes H Hegemann

**Affiliations:** Lehrstuhl für Funktionelle Genomforschung der Mikroorganismen, Heinrich-Heine-Universität 40225 Düsseldorf, Germany.

## Abstract

*Chlamydia pneumoniae*, an obligate intracellular human pathogen, causes a number of respiratory diseases. We explored the role of the conserved OmcB protein in *C. pneumoniae* infections, using yeast display technology. (i) Yeast cells presenting OmcB were found to adhere to human epithelial cells. (ii) Pre-incubation of OmcB yeast cells with heparin, but not other glycosaminoglycans (GAGs), abrogated adhesion. (iii) Pre-treatment of the target cells with heparinase inhibited adherence, and GAG-deficient CHO cell lines failed to bind OmcB yeast. (iv) A heparin-binding motif present near the N-terminus of OmcB is required for host cell binding. (v) Pre-treatment of chlamydial elementary bodies (EBs) with anti-OmcB antibody or pre-incubation of target cells with recombinant OmcB protein reduced infectivity upon challenge with *C. pneumoniae.* (vi) Adhesion of fluorescently labelled EBs to epithelial or endothelial cells was abrogated by prior addition of heparin or OmcB protein. Thus, *C. pneumoniae* OmcB is an adhesin that binds heparan sulphate-like GAGs. OmcB from *Chlamydia trachomatis* serovar L1 also adheres to human cells in a heparin-dependent way, unlike its counterpart from serovar E. We show that a single position in the OmcB sequence determines heparin dependence/independence, and variations there may reflect differences between the two serovars in cell tropism and disease pattern.

## Introduction

Chlamydia cause widespread infections in humans, other mammals and birds. *Chlamydia trachomatis*, a pathogen of mucosal surfaces, is one of the leading causes of sexually transmitted disease and the most common cause of preventable blindness worldwide (Schachter, 1999; Byrne and Ojcius, 2004). *Chlamydia psittaci* infects birds and occasionally causes psittacosis in humans, while *C. pneumoniae* was first isolated as a respiratory pathogen. Exposure to *C. pneumoniae* is very common and the prevalence of infection increases with age (Grayston, 2000). *C. pneumoniae* infections cause approximately 10% of community-acquired pneumonia and 5% of bronchitis and sinusitis cases (Grayston, 1992). In addition, *C. pneumoniae* infections have been implicated in the aetiology of stroke, atherosclerosis, Alzheimer's disease and multiple sclerosis (Saikku, 2000; Lindsberg and Grau, 2003; Mussa *et al.*, 2006; Stratton and Wheldon, 2006). Evidence for an association between *C. pneumoniae* and atherogenesis stems from the fact that these bacteria have been detected in atherosclerotic plaques and isolated in culture from coronary arteries (Kuo *et al.*, 1993; Maass *et al.*, 1998). Furthermore, chlamydia are also associated with various chronic diseases, ranging from reactive arthritis to asthma (Kuo *et al.*, 1995; Von Hertzen, 2002; Zeidler *et al.*, 2004). It is not clear whether persistence of an unresolved chlamydial infection or repeated chlamydial infections are relevant for these chronic diseases (Hogan *et al.*, 2004).

Chlamydia are pathogenic, Gram-negative, obligate intracellular eubacteria with a unique biphasic developmental cycle involving two different cell forms (Moulder *et al.*, 1984; Abdelrahman and Belland, 2005). Infectious but metabolically inert elementary bodies (EBs) adhere to eukaryotic host cells, stimulating their own uptake. Internalized EBs remain in an intracellular compartment termed an inclusion, where they differentiate into metabolically active but non-infectious reticulate bodies (RBs), which divide by binary fission. After 9–11 generations the RBs re-differentiate into EBs, which are then released from the host cell to start a new round of infection (reviewed in Abdelrahman and Belland, 2005).

Bacterial infection is a multifactorial process that can be divided into several distinct stages. Bacterial adherence to host cell surfaces is the first step in the establishment of an infection and is a prerequisite for all subsequent events. At this early stage of infection the specificity of the pathogen–host cell interaction is determined by bacterial surface proteins (adhesins) and receptors on the host cell surface. These specific interactions may also help determine the cell and tissue tropism of the bacteria (Finlay and Falkow, 1989; Boyle and Finlay, 2003; Pizarro-Cerda and Cossart, 2006). Glycosaminoglycan (GAG) structures on the mammalian cell surface play an important role in interactions with many microbial pathogens, and are recognized by a number of viral, bacterial and protozoan adhesins (Rostand and Esko, 1997; Wadstrom and Ljungh, 1999; Menozzi *et al.*, 2002). Chlamydia can infect a variety of non-phagocytic animal cell types, indicating that the bacterial adhesins recognize conserved cellular receptors and/or that EB attachment is mediated by a variety of different adhesin–receptor combinations (reviewed in Hackstadt, 1999; Abdelrahman and Belland, 2005; Dautry-Varsat *et al.*, 2005; Campbell and Kuo, 2006). Despite the essential role of the adhesion process, we have only limited knowledge of the nature of the chlamydial adhesins and their receptors (Dautry-Varsat *et al.*, 2005; Campbell and Kuo, 2006). A number of studies have implied that GAGs are required for the initial phase of the chlamydial EB attachment process (Zhang and Stephens, 1992; Chen and Stephens, 1994; 1997; Zaretzky *et al.*, 1995; Chen *et al.*, 1996; Davis and Wyrick, 1997; Gutierrez-Martin *et al.*, 1997). Thus, the addition of exogenous heparan sulphate, or the heparan sulphate analogue heparin, inhibits infection by *C. trachomatis*, *C. psittaci* and *C. pneumoniae*, although the degree of inhibition observed is dependent on the chlamydial species, biovar, isolate and human cell line used (Chen and Stephens, 1997; Gutierrez-Martin *et al.*, 1997; Taraktchoglou *et al.*, 2001; Wuppermann *et al.*, 2001; Yabushita *et al.*, 2002; Beswick *et al.*, 2003; Darville *et al.*, 2004; Yan *et al.*, 2006). Specifically, comparative studies of the two *C. trachomatis* biovars, the trachoma biovar (serovars A through K) and the lymphogranuloma venereum (LGV) biovar (serovars L1, L2 and L3), which differ in their infection characteristics and invasiveness in clinical disease, display different GAG dependencies (reviewed in Campbell and Kuo, 2006). While heparan sulphate blocks binding of the LGV biovar to both non-polarized and polarized human epithelial cells, binding of serovar B or E EBs is only partly inhibited or even unaffected (Chen and Stephens, 1997; Davis and Wyrick, 1997; Taraktchoglou *et al.*, 2001). This suggests that the more invasive LGV biovars, which are primarily submucosal pathogens, may use heparan sulphate as an adhesin to colonize basolateral domains of the mucosal epithelium, while serovar E may use other GAGs (Campbell and Kuo, 2006). For *C. trachomatis* a trimolecular mechanism has been proposed, in which GAG-like structures serve as a bridge between an adhesin on the EB and a receptor on the host cell surface (Zhang and Stephens, 1992; Rasmussen-Lathrop *et al.*, 2000; Stephens *et al.*, 2006). For *C. pneumoniae* it has been shown that binding of EBs to heparin sulphate-like GAGs on the host cell surface is a prerequisite for subsequent infection (Wuppermann *et al.*, 2001). More recently it was reported, for other *C. pneumoniae* strains and other eukaryotic cell lines, that GAGs on the surfaces of both target cells and EBs may play a role in invasion (Beswick *et al.*, 2003; Yan *et al.*, 2006). The role of heparan sulphate-like GAGs in infection by *C. pneumoniae* is also supported by the fact that the induction of pro-inflammatory cytokine gene expression by a *C. pneumoniae* infection depends on adherence of the bacteria to human lung epithelial cells and can be blocked by addition of heparin (Yang *et al.*, 2003).

Indeed, the GAG-binding outer membrane protein OmcB from *C. trachomatis* L2 was identified by heparin affinity chromatography, although its role in the chlamydial adhesion process and its relevance for infection remained unknown (Stephens *et al.*, 2001). OmcB was originally identified as a major component of the chlamydial outer membrane complex, and is conserved among all chlamydial species (Wagels *et al.*, 1994; Hatch, 1999). OmcB has been variously localized to the periplasm or the outer membrane of EBs (Watson *et al.*, 1994; Everett and Hatch, 1995; Ting *et al.*, 1995; Mygind *et al.*, 1998; Stephens *et al.*, 2001). However, during chlamydial infections OmcB induces a strong antibody response, suggesting that it is readily accessible to the humoral immune response, which suggests a surface localization for it (Wagels *et al.*, 1994; Mygind *et al.*, 1998; Cunningham and Ward, 2003; Klein *et al.*, 2003; Portig *et al.*, 2003).

The absence of a genetic system for chlamydia makes the identification and functional characterization of chlamydial proteins involved in the adhesion process particularly difficult. To overcome these limitations we have used a new test system for the functional characterization of adhesion proteins. Display of heterologous proteins on the surface of phages or microorganisms like *Escherichia coli*, Gram-positive bacteria or yeast has proven to be extremely useful in a number of experimental settings, such as the development of a live vaccine, design of whole-cell biocatalysts or screening of antibody libraries (Georgiou *et al.*, 1997; Kondo and Ueda, 2004; Jostock and Dubel, 2005; Mullen *et al.*, 2006). Yeast surface display technology offers the additional advantages that (i) all cloning steps are readily performed by homologous recombination in yeast and (ii) the size of the protein to be presented is not a limitation (Boder and Wittrup, 1997; Swers *et al.*, 2004). In the yeast display system yeast cells express a fusion protein comprising the a-agglutinin protein Aga2 linked to the protein of interest; this is then secreted and binds via disulphide bridges to the cell wall protein Aga1 (Boder and Wittrup, 1997).

In order to study the function of the *C. pneumoniae* OmcB protein we established a yeast adhesion system in which the binding of live OmcB-presenting yeast cells to human cells could be studied. Here we show that the OmcB protein from *C. pneumoniae* mediates adhesion to human epithelial HEp-2 cells. OmcB adhesion is dependent on the N-terminal domain of the protein and a heparin-binding motif present in this region. Infection inhibition experiments using recombinant *C. pneumoniae* OmcB protein or OmcB antibodies reveal that OmcB is essential for infection, and specifically is involved in the adhesion of the infectious EBs to the target cells. The ability of OmcB proteins to bind to human cells seems to be a general property of chlamydia, as OmcB from the *C. trachomatis* serovars L1 and E (and from *Chlamydia caviae*) adheres to HEp-2 cells. Intriguingly binding of the OmcB protein from *C. trachomatis* serovar E to HEp-2 cells is completely independent of heparin, suggesting differences in the mode of host cell invasion *in vivo*. Our mutational analysis of the OmcB proteins from *C. trachomatis* L1 and E indicates that binding can be switched from heparin independence to heparin dependence by changing a single amino acid.

## Results

### The yeast display system identifies *C. pneumoniae* OmcB as an adhesion protein

**Fig. 1:**
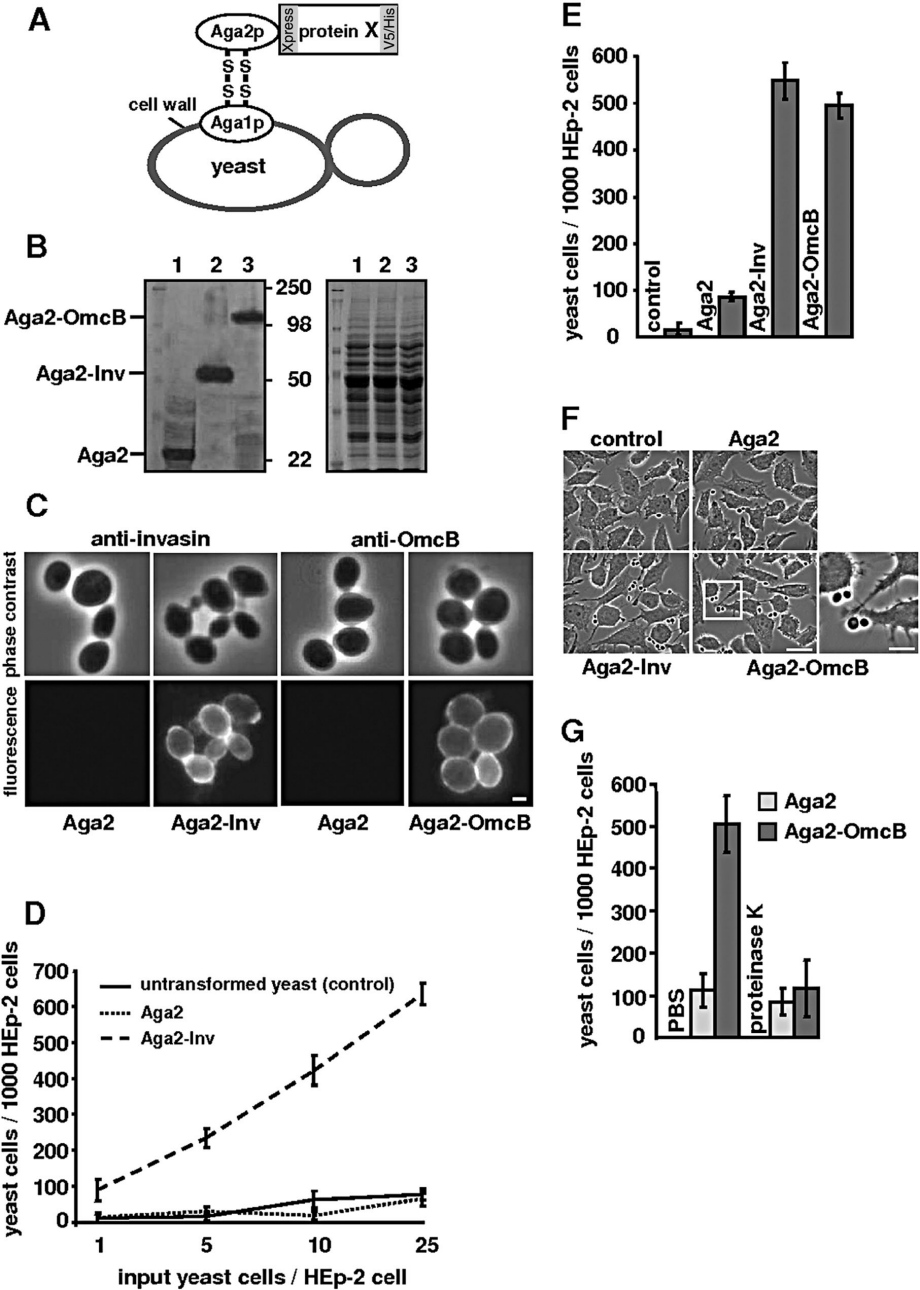
Yeast cells expressing Aga2–Inv or Aga2–OmcB adhere to human cells. A. Schematic representation of the yeast display system. The protein of interest is presented on the yeast cell surface as a consequence of its fusion to Aga2p. B. Western analysis of Aga2 (lane 1), Aga2–Inv (lane 2) and Aga2–OmcB (lane 3). Left: Protein extracts from strains expressing Aga2, Aga2–Inv or Aga2–OmcB were digested with α-mannosidase to remove Aga2 O-glycosylation, resolved by SDS-PAGE and probed with an anti-His antibody. Right: Coomassie-stained SDS-PAGE of the protein extracts shown in the left panel. The positions of the size markers (250, 98, 50 and 22 kDa) are indicated. C. Detection of Aga2 fusion proteins on the yeast cell surface. Yeast cells expressing Aga2, Aga2–Inv or Aga2–OmcB were fixed and stained with anti-invasin or anti-OmcB antibodies for visualization of Aga2–Inv and Aga2–OmcB proteins respectively. Bar 1 μm. D–G. Adhesion of yeast cells to HEp-2 cells. (D) Varying numbers of untransformed yeast cells or yeast cells expressing Aga2 or Aga2–Inv from a plasmid were incubated with 1 × 10^5^ HEp-2 cells, and the number of yeast cells associated with HEp-2 cells was determined by microscopy. (*n* = 1000 HEp-2 cells, No. experiments = 4, *P* < 0.0001). (E) Schematic representation of the numbers of yeast control cells or yeast cells expressing Aga2, Aga2–Inv and Aga2–OmcB adhering to 1000 HEp-2 cells. (*n* = 1000 HEp-2 cells, No. experiments = 4, *P* < 0.0001). (F) Photomicrographs of HEp-2 cells after incubation with yeast cells expressing the indicated proteins. Bar 10 μm. The cells indicated by the white box are shown enlarged in the rightmost panel. Bar 5 μm. (G) Adhesion of Aga2 or Aga2–OmcB-expressing yeast cells to HEp-2 cells. Yeast cells (1 × 10^6^) were treated with PBS or proteinase K (4 mg ml^−1^) for 1 h at 37°C, washed three times and then added to HEp-2 cells (*n* = 1000 HEp-2 cells, No. experiments = 4, *P* < 0.0001).

The adhesion of *C. pneumoniae* to host cells is the first and crucial step in bacterial pathogenesis. As this pathogen cannot be genetically manipulated, we used the heterologous yeast display system to study the adhesion properties of chlamydial proteins. In this system, *Saccharomyces cerevisiae* cells express heterologous proteins on their surfaces (Fig. 1A). The protein of interest is expressed as a fusion protein with the yeast a-agglutinin receptor subunit Aga2, which is anchored to the yeast cell wall via its interaction with the resident Aga1 subunit (Fig. 1A) (Boder and Wittrup, 1997). Expression of Aga2 and Aga2 fusion proteins occurs via the galactose-inducible *GAL1* promoter on a plasmid (Boder and Wittrup, 1997). We expressed Aga2, Aga2 fused to the adhesion domain [amino acid (aa) 790 to aa 986] of the *Yersinia pseudotuberculosis* invasin (Dersch and Isberg, 1999), referred to here as Aga2–Inv, and Aga2–OmcB (*C. pneumoniae* OmcB) in yeast. All three proteins were expressed in similar amounts (Fig. 1B), and Aga2–Inv and Aga2–OmcB could be detected on the yeast cell surface with anti-invasin and anti-OmcB antibodies respectively (Fig. 1C).

To test whether the yeast display system was suitable for the identification of bacterial adhesins, we incubated various numbers of yeast cells expressing the well-defined invasin adhesion domain (aa 790–986) with 1 × 10^5^ HEp-2 cells seeded on glass coverslips. In contrast to untransformed yeast cells or yeast cells expressing Aga2, yeast cells presenting the Aga2–Inv fusion protein on the cell surface showed a high affinity for HEp-2 cells (Fig. 1D). In particular, we found that the ratio of Aga2–Inv yeast cells to HEp-2 cells correlated directly with the number of yeast cells adhering to the human cells (Fig. 1D). This was not observed for untransformed yeast cells or Aga2-expressing yeast cells (Fig. 1D). Thus, the yeast display system is indeed a suitable model with which to study the adhesion properties of bacterial proteins.

Microscopic inspection revealed that Aga2–Inv yeast cells mainly bound laterally to HEp-2 cells and bud-forming yeast cells showed uniform binding via the mother cell and the bud (data not shown). Invasin-coated latex beads not only adhere to human cells but are also taken up by them (Dersch and Isberg, 1999). However, when we performed our yeast adhesion experiments at 37°C uptake of the invasin-coated yeast cells was not observed (data not shown).

Next, we tested if the *C. pneumoniae* OmcB protein had adhesive properties by analysing the ability of Aga2–OmcB-expressing yeast cells to adhere to HEp-2 cells. We found that Aga2–OmcB yeast cells were able to adhere to HEp-2 cells just like Aga2–Inv yeast cells (Fig. 1E and F). Proteolytic degradation of the surface-expressed Aga2–OmcB fusion protein by proteinase K or trypsin led to a complete loss of adhesion (Fig. 1G, data not shown). Thus, OmcB mediates adhesion of yeast cells to HEp-2 cells.

### The *C. pneumoniae* OmcB binds to heparan sulphate-like structures on host cells

**Fig. 2:**
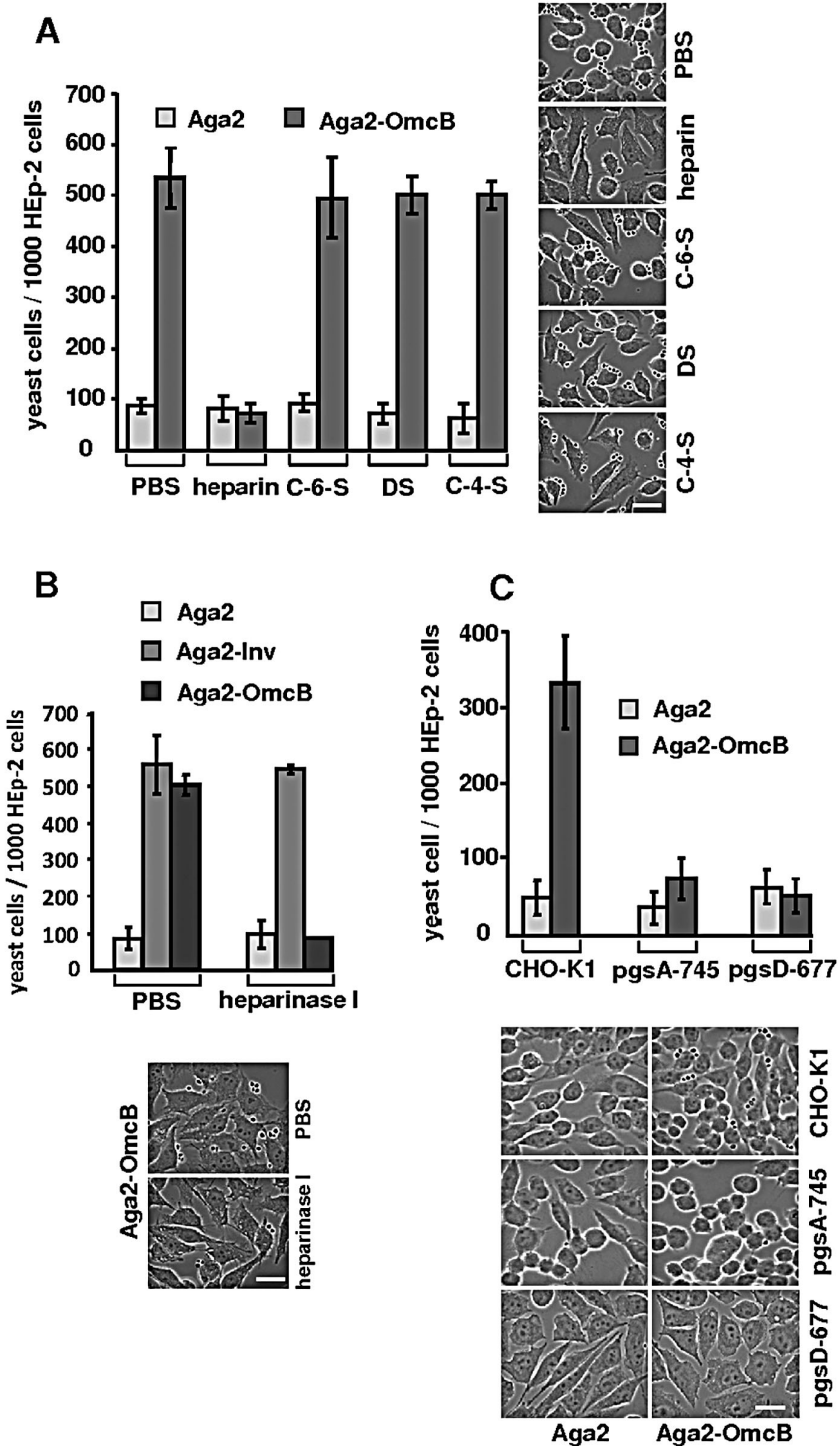
Aga2–OmcB adhesion is dependent on host cell GAGs. A. Left: Aga2- or Aga2–OmcB-expressing yeast cells were incubated with 500 μg ml^−1^ of the soluble GAGs heparin, chondroitin-6-sulphate (C-6-S), dermatan sulphate (DS) or chondroitin-4-sulphate (C-6-S) prior to the adhesion assay and the numbers of yeast cells adhering to human cells were determined by microscopy. Yeast cells treated with PBS served as controls (*n* = 1000 HEp-2 cells, No. experiments = 4, *P* < 0.0001). Right: Photomicrographs of GAG-treated Aga2–OmcB-expressing yeast cells adhering to human cells. Bar 10 μm. B. Effects of treatment with heparinase on the ability of HEp-2 cells to bind Aga2-, Aga2–Inv- and Aga2–OmcB-expressing yeast cells. HEp-2 cells were incubated with PBS or 50 U heparinase I prior to the adhesion experiment. The photomicrographs show adhesion of Aga2–OmcB-expressing yeast cells to HEp-2 cells. C. Efficiency of association of Aga2- or Aga2–OmcB-expressing yeast cells with wild-type (CHO-K1) or GAG-deficient (pgsA-745 and pgsD-677) cell lines is plotted diagrammatically and illustrated in the photomicrographs. Bar 10 μm (*n* = 1000 cells, No. experiments = 4, *P* < 0.01).

Adhesion of, and subsequent infection by, *C. pneumoniae* is dependent on GAGs such as heparan sulphate present on the host cell (Davis and Wyrick, 1997; Wuppermann *et al.*, 2001; Beswick *et al.*, 2003). To test if GAGs also play a role in the adhesion of *C. pneumoniae* mediated by OmcB, we pre-incubated Aga2- and Aga2–OmcB-expressing yeast cells with various soluble GAG derivatives prior to the adhesion assays. Pre-incubation with heparin, a commonly used analogue for heparan sulphate, rendered Aga2–OmcB cells unable to adhere to HEp-2 cells (Fig. 2A). Other GAGs, such as chondroitin-6-sulphate (C-6-S), dermatan sulphate (DS) or chondroitin-4-sulphate (C-4-S), did not influence the binding of Aga2–OmcB yeast to HEp-2 cells (Fig. 2A). Thus OmcB specifically binds heparin-like GAGs.

Further support for the idea that OmcB binds heparin-like structures comes from our observation that enzymatic removal of the heparan sulphate structures from the surface of HEp-2 cells strongly inhibited binding of Aga2–OmcB yeast cells, while adhesion of Aga2–Inv yeast cells to HEp-2 cells was unaffected by treatment of the latter with heparinase I (Fig. 2B). Our finding that OmcB present on yeast cells binds to heparan sulphate was also supported by the results of yeast adhesion assays using the CHO mutant cell lines pgsA-745 and pgsD-677 as targets (Fig. 2C). These cell lines are defective in GAG biosynthesis (Esko *et al.*, 1985). The pgsA-745 line is unable to synthesize any GAGs, while pgsD-677 cannot synthesize heparan sulphate but accumulates chondroitin sulphate. Aga2–OmcB yeast cells were able to adhere to wild-type CHO-K1 cells, but were unable to associate with pgsA-645 or pgsD-677 cells (Fig. 2C). These results confirm that OmcB binds heparan sulphate-like GAGs on human cells.

### The N-terminal part of OmcB is required for adhesion

**Fig. 3:**
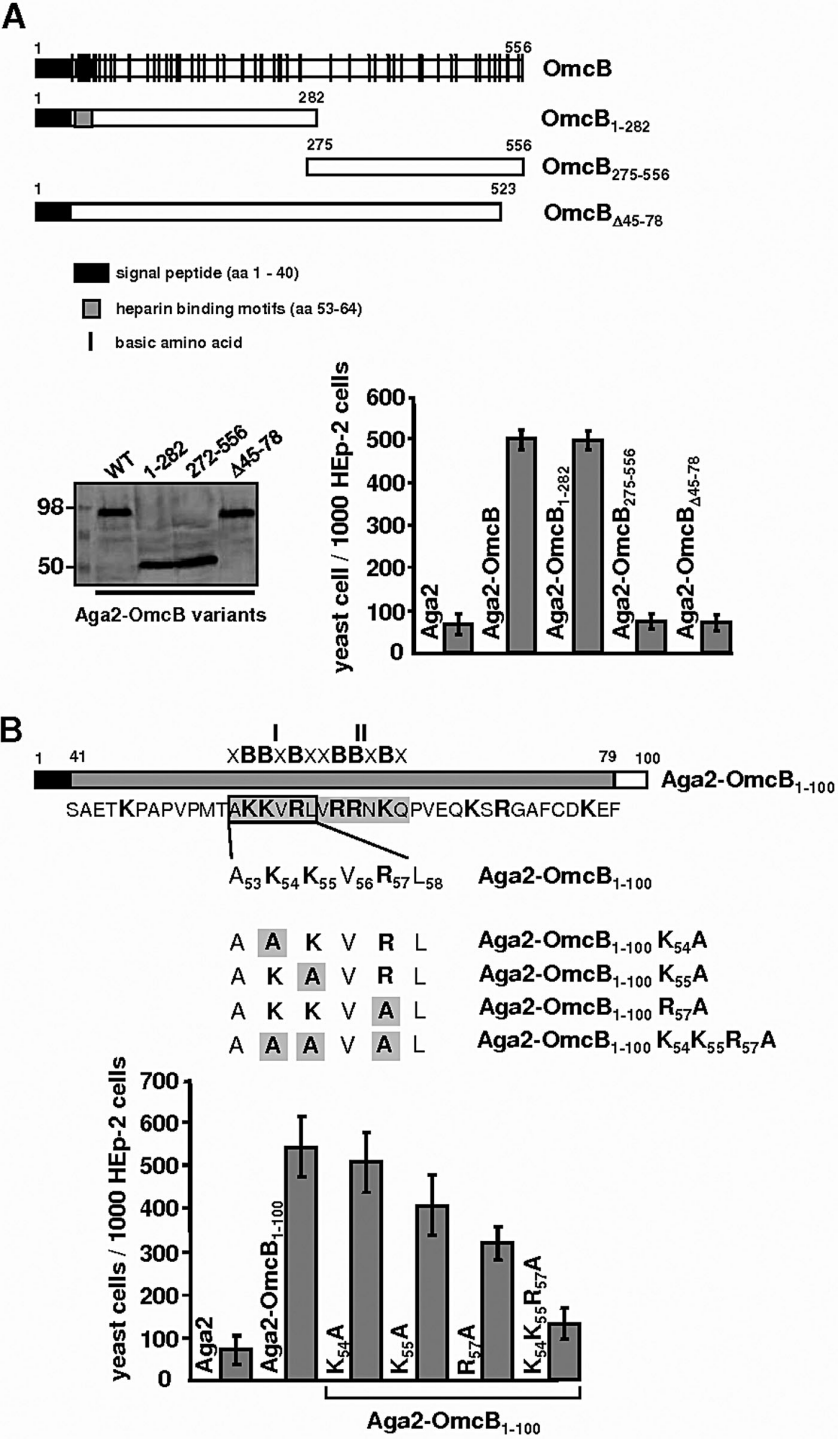
OmcB protein variants and adhesion. A. Top: Schematic representations of the wild-type OmcB protein from *C. pneumoniae*, and the deletion derivatives used here, with basic amino acids (black bars), signal peptide sequence (black box) and heparin-binding motifs (grey box) indicated. Bottom left: Western blot analysis of protein extracts from strains expressing Aga2–OmcB_1−282_, Aga2–OmcB_275−556_ and Aga2–OmcB_Δ45−78_. After digestion with α-mannosidase, cell extracts were resolved by SDS-PAGE and probed with an anti-His antibody. Bottom right: Adhesion of yeast cells expressing Aga2, Aga2–OmcB_1−282_, Aga2–OmcB_275−556_ or Aga2–OmcB_Δ45−78_ to HEp-2 cells. (*n* = 1000 HEp-2 cells, No. experiments = 4, *P* < 0.0001). B. Schematic representation of the amino acid changes generated in the OmcB variant Aga2–OmcB_1−100_. The heparin-binding motifs I and II are indicated. These variants show reduced adherence to HEp-2 cells (bottom diagram) (*n* = 1000 HEp-2 cells, No. experiments = 4, *P* < 0.0001).

To identify the domain of the *C. pneumoniae* OmcB that mediates binding to human cells we generated truncated versions of the protein and tested them in the yeast display system. Western blot analysis revealed that the truncation mutants are produced in similar amounts to the full-length protein (Fig. 3A). A C-terminal OmcB deletion variant, Aga2–OmcB_1−282_, behaved like wild-type OmcB, while the N-terminal deletion variant Aga2–OmcB_275−556_ showed no adhesive properties (Fig. 3A). Thus the N-terminal part of OmcB harbours the binding domain required for adhesion.

This portion of the protein has a large number of basic amino acids, particularly in the stretch between amino acids 41 and 79. Basic amino acids have been shown to be important for the binding of proteins to GAGs (Cardin and Weintraub, 1989; Hileman *et al.*, 1998; Stephens *et al.*, 2001). We therefore created the OmcB deletion variant OmcB_Δ45−78_, which lacks amino acids 45–78, and tested this variant in our yeast adhesion assay. Yeast cells expressing Aga2–OmcB_Δ45−78_ were unable to adhere to HEp-2 cells (Fig. 3A), implying that the basic region plays an important role in the adhesion process. A sequence comparison of OmcB homologues from various chlamydial species revealed that this region of the *C. pneumoniae* OmcB protein harboured two putative heparin-binding motifs (amino acids 53–58 and 59–64 respectively) with the consensus sequence XBBXBX (X = hydropathic amino acid, B = basic amino acid) (Hileman *et al.*, 1998) (Fig. 3B). The first motif is conserved among OmcB proteins from all chlamydia, with the exception of environmental Parachlamydia. To test whether such a heparin-binding motif was required for adhesion, we replaced several of the basic amino acids found in heparin-binding motif I with alanines, and tested the ability of the mutant protein to function in our yeast adhesion assay. All mutations were generated in the N-terminal variant OmcB_1−100_. Yeast cells expressing Aga2–OmcB and Aga2–OmcB_1−100_ display comparable levels of adhesion to HEp-2 cells, indicating that the OmcB_1−100_ segment is mainly responsible for adhesion (Fig. 3A and B). Using site-directed mutagenesis, we generated OmcB_1−100_ variants with single-amino-acid changes at positions 54 (lysine to alanine), 55 (lysine to alanine) and 57 (arginine to alanine) respectively (Fig. 3B). Substitution of the lysine residues decreased adhesion by 5% or 10% (OmcB_1−100_K_54_A and OmcB_1−100_K_55_A respectively) compared with wild-type OmcB_1−100_. Replacement of the arginine residue reduced adhesion by 35% (Fig. 3B). When all three mutations were combined in the variant OmcB_1−100_K_54_K_55_R_57_A adhesion was completely abolished (Fig. 3B). Thus heparin-binding motif I is required for adhesion, and the basic amino acids present in this sequence all contribute to this function, although to varying degrees.

**Fig. 4:**
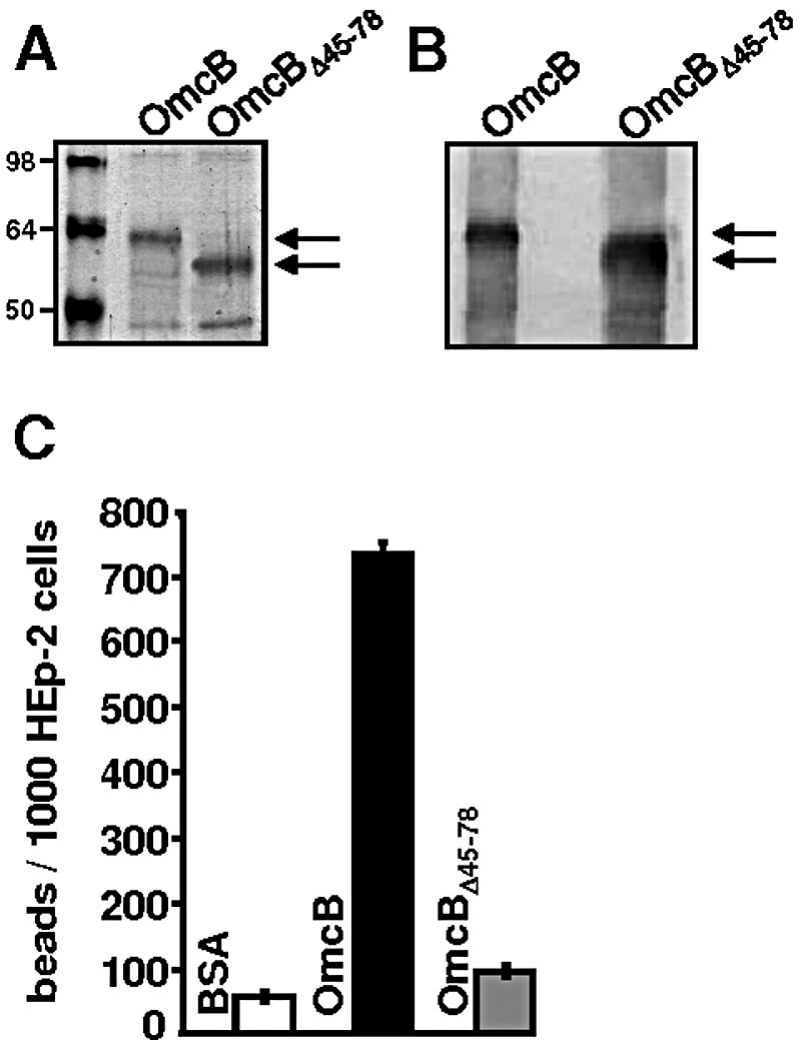
Adhesion of OmcB-coated latex beads to human cells. A. Affinity-purified, recombinant wild-type OmcB and OmcB_Δ45−78_ proteins (black arrows) were resolved by SDS-PAGE and stained with Coomassie. Bands of higher molecular weight represent contaminants, and bands of lower molecular weight are degradation products. Positions of size markers (98, 64 and 50 kDa) are indicated. B. Equal amounts of beads coated with OmcB or OmcB_Δ45−78_ protein (black arrows) were separated by SDS-PAGE and probed with an anti-His antibody. C. Adhesion of coated latex beads to HEp-2 cells. Latex beads (1 × 10^6^) coated with 100 μg of BSA, OmcB or OmcB_Δ45−78_ were incubated with 1 × 10^5^ HEp-2 cells and the numbers of beads associated with HEp-2 cells were determined by microscopy. (*n* = 1000 HEp-2 cells, No. experiments = 4, *P* < 0.0001).

In an effort to confirm the results obtained with the yeast display system, we employed the well-established latex bead assay (Dersch and Isberg, 1999). To this end, full-length OmcB and OmcB_Δ45−78_ were expressed in and purified from *E. coli* (Fig. 4A). Similar amounts of the purified OmcB variants were coupled to latex beads (Fig. 4B), which were then tested for the ability to adhere to HEp-2 cells. We found that beads coated with the wild-type OmcB were able to adhere to HEp-2 cells, while beads bearing OmcB_Δ45−78_ could not (Fig. 4C). In addition, increasing the amounts of OmcB attached to beads enhanced their ability to associate with HEp-2 cells (Fig. S1). As expected adhesion of beads coated with the wild-type OmcB was sensitive to heparin (data not shown).

### The *C. pneumoniae* OmcB is localized to the surfaces of EBs and RBs in infected HEp-2 cells

**Fig. 5:**
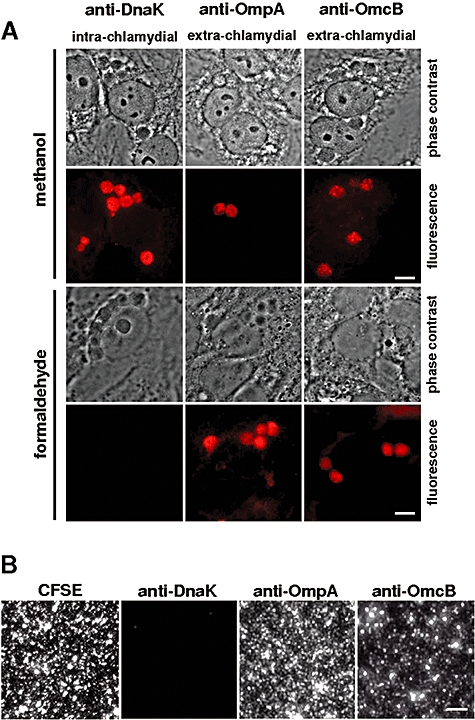
Localization of *C. pneumoniae* OmcB. A. Photomicrographs of HEp-2 cells infected with *C. pneumoniae* at moi 1 in the presence of 1.2 μg ml^−1^ cycloheximide for 48 h. Cells were fixed with methanol to visualize intra- and extrachlamydial antigens by staining with anti-DnaK, anti-OmpA and anti-OmcB antibodies. Fixation with formaldehyde and permeabilization with 0.5% Triton X-100 was used to visualize extrachlamydial antigens (Birkelund *et al.*, 1990). Bar 10 μm. B. Left panel: Photomicrograph of purified, non-fixed *C. pneumoniae* EBs stained with CFSE. Right panels: Equal amounts of purified, non-fixed *C. pneumoniae* EBs were used for microimmunofluorescence (MIF) analysis. Chlamydial antigens were detected using anti-DnaK, anti-OmpA and anti-OmcB antibodies. Bar 1 μm.

In order to act as an adhesin, OmcB must be exposed on the surface of the bacterial cell. To investigate the localization of OmcB, indirect immunofluorescence microscopy was performed on HEp-2 cells infected with *C. pneumoniae* 48 h post infection. In methanol-fixed cells an antibody directed against the intrachlamydial DnaK protein, as well as antibodies directed against the extrachlamydial OmpA and OmcB proteins, reacted with the bacteria in the inclusion (Fig. 5A). In formaldehyde-fixed cells, cross-linking of outer membrane proteins renders intrachlamydial proteins inaccessible to the antibody. Indeed the anti-DnaK antibody did not react with formaldehyde-treated bacteria, but both anti-OmpA and anti-OmcB antibodies did, indicating that they are present on the bacterial cell surface (Fig. 5A). Similar experiments on unfixed *C. pneumoniae* EBs confirmed that both OmcB and OmpA were surface-localized, whereas the DnaK epitopes were not accessible to antibodies (Fig. 5B).

### The OmcB protein mediates attachment and subsequent infection of *C. pneumoniae*

**Fig. 6:**
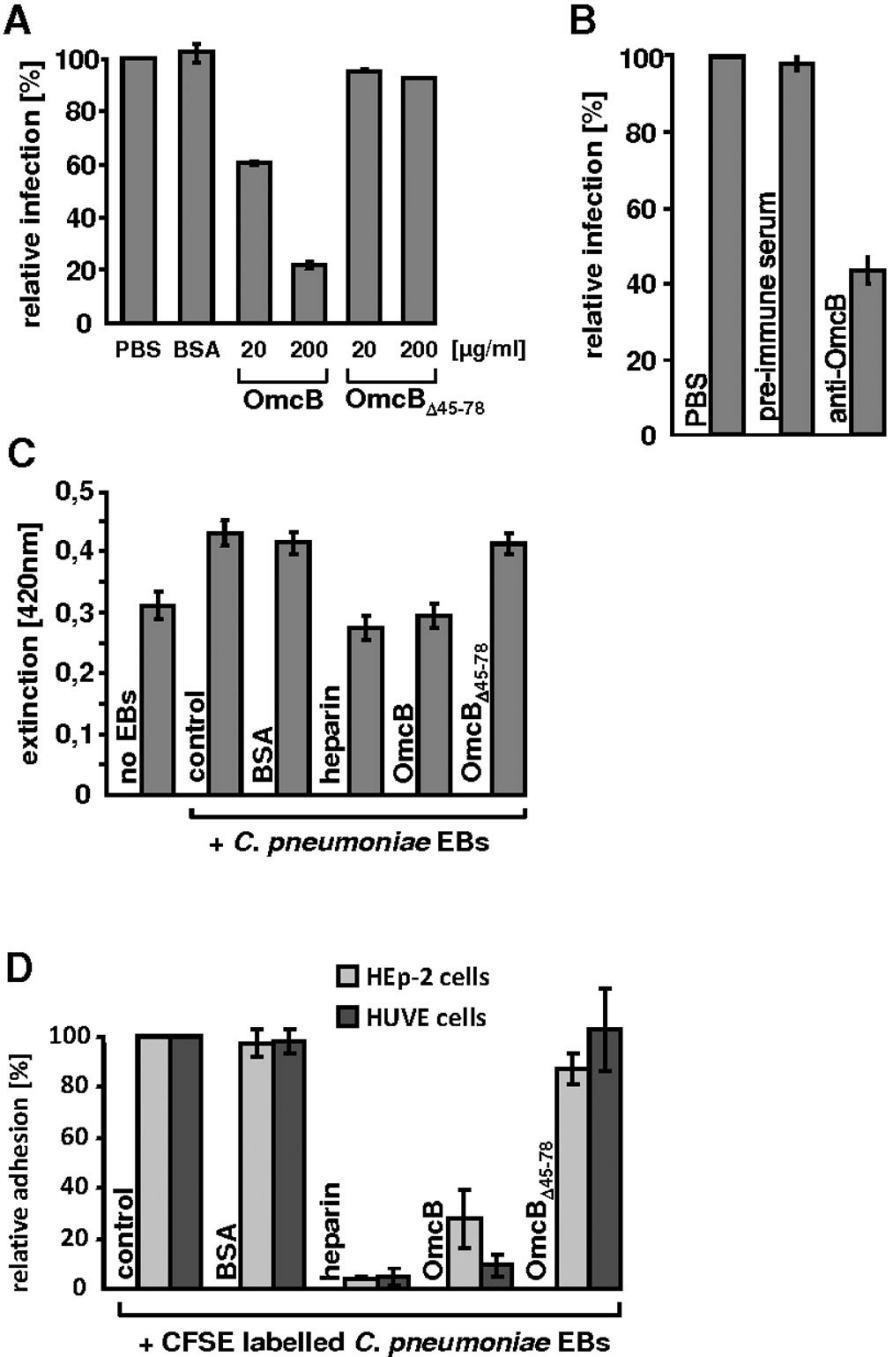
Inhibition of infection by *C. pneumoniae*. A and B. *C. pneumoniae* infection is inhibited by addition of recombinant OmcB protein or an OmcB-specific antibody. (A) HEp-2 cells (1 × 10^6^) were incubated with PBS, 200 μg of BSA, or 20 μg or 200 μg of recombinant OmcB or OmcB_Δ45−78_ protein prior to challenge with purified *C. pneumoniae* EBs. Cells were fixed at 48 h post infection and the numbers of inclusions determined by microscopy. The number of inclusions found in the PBS control was set to 100%. (*n* = 20 microscopic fields, No. experiments = 4, *P* < 0.0001). (B) Purified *C. pneumoniae* EBs were incubated with PBS, pre-immune serum or anti-OmcB antibody before being exposed to HEp-2 cells. The numbers of inclusions formed was determined by microscopy 48 h post infection. (*n* = 20 microscopic fields, No. experiments = 4, *P* < 0.0001). C. Binding of purified *C. pneumoniae* EBs to HEp-2 cells was detected by a colorimetric assay using a chlamydia-specific primary antibody and a HRP-coupled secondary antibody. Attachment of *C. pneumoniae* EBs in the presence of PBS or 200 μg of BSA served as controls, and these data were compared with samples to which heparin (500 μg ml^−1^) or recombinant OmcB or OmcB_Δ45−78_ protein (200 μg) had been added. (*n* = 2 wells, No. experiments = 6, *P* < 0.01). D. Adhesion of viable, CFSE-stained *C. pneumoniae* EBs to HEp-2 or human umbilical vein endothelial cells (HUVEC) was detected by flow cytometric analysis. The same amounts of protein and heparin as in (C) were used in this experiment.

Our data strongly indicate that the *C. pneumoniae* OmcB protein is required for adhesion of the bacteria to HEp-2 cells in a GAG-dependent manner. This implies that OmcB plays a part in the infection process. We therefore tested whether prior addition of recombinant OmcB protein to HEp-2 cells interfered with infection by *C. pneumoniae*. Incubation of HEp-2 cells with various amounts of recombinant OmcB (20–200 μg ml^−1^) strongly inhibited subsequent infection by *C. pneumoniae* in a dose-dependent manner (Fig. 6A). In contrast, prior incubation of HEp-2 cells with the OmcB_Δ45−78_ variant had no influence on infection (Fig. 6A). Next, we blocked OmcB function by incubating purified chlamydial EBs with an anti-OmcB antibody. Incubation of chlamydial EBs with pre-immune serum did not decrease the incidence of infection. However, prior addition of the anti-OmcB antibody reduced infectivity by 60% (Fig. 6B).

To demonstrate directly that OmcB is an adhesin that is essential for the attachment of *C. pneumoniae* to its target cells, ELISA-based attachment assays and flow cytometric analyses were employed. In the ELISA assay we measured the binding of purified chlamydial EBs to epithelial HEp-2 cells (Fig. 6C). As expected, heparin-treated controls showed significantly less *C. pneumoniae* binding, in agreement with the previously described effect of heparin (Wuppermann *et al.*, 2001). Presence of recombinant OmcB protein led to a significant decrease in EB attachment to HEp-2 cells, whereas addition of OmcB_Δ45−78_ protein had no inhibitory effect (Fig. 6C). This result was confirmed by the cytometric analysis of binding of CFSE-labelled EBs to epithelial and endothelial cells. The presence of recombinant OmcB protein led to a 70% and a 91% decrease in EB attachment to HEp-2 and HUVE cells, respectively, whereas addition of OmcB_Δ45−78_ protein had no inhibitory effect (Fig. 6D). We therefore concluded that the recombinant OmcB protein occupies the GAG binding site on epithelial and endothelial host cells, thus inhibiting adhesion of EBs and subsequent infection.

### Variations in the amino acid sequence of OmcB correlate with GAG-dependent adhesion

Glycosaminoglycan-dependent infection has been observed with various chlamydial species, such as *C. pneumoniae, C. caviae* and *C. trachomatis* serovars LGV (L1, L2), whereas infection with the *C. trachomatis* serovars B and E appears to be rather independent of GAG (Chen and Stephens, 1994; 1997; Gutierrez-Martin *et al.*, 1997; Taraktchoglou *et al.*, 2001; Wuppermann *et al.*, 2001; Beswick *et al.*, 2003).

**Fig. 7:**
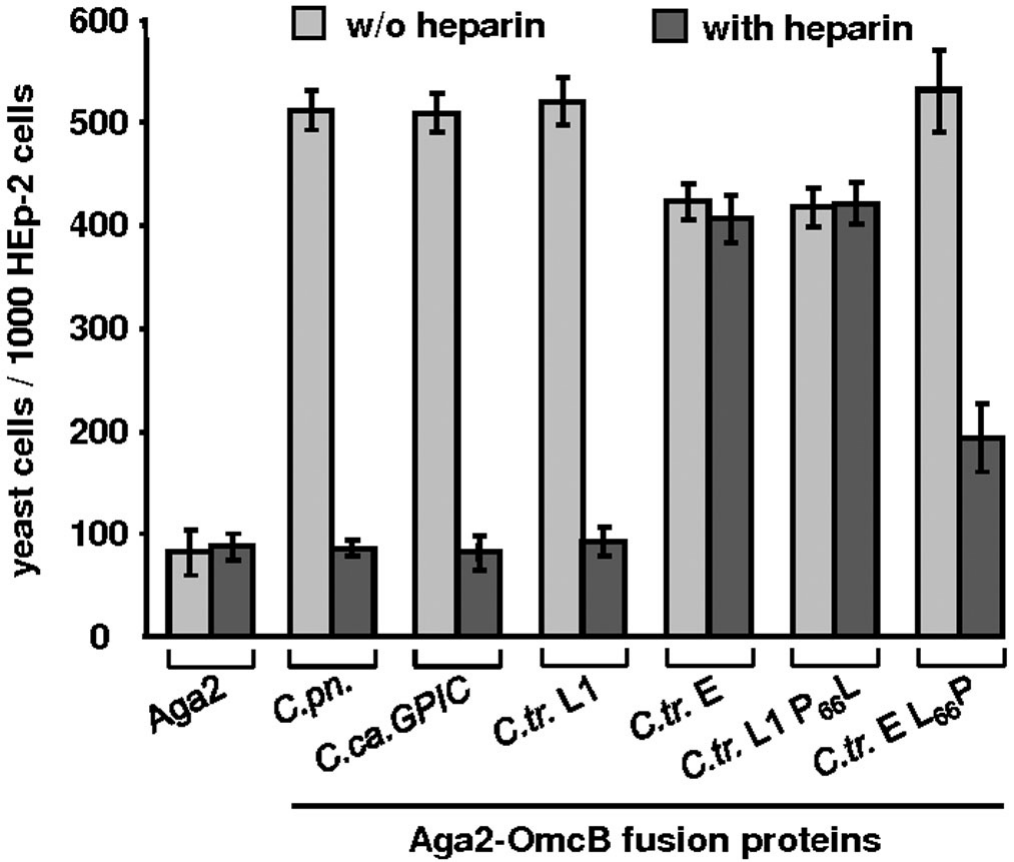
Assay of the GAG dependency of binding by *C. caviae* OmcB and *C. trachomatis* OmcB variants. Schematic representation of the adhesion of various yeast transformants to HEp-2 cells. Yeast cells expressing Aga2, Aga2–OmcB-*C.pn*., Aga2–OmcB-*C.ca.* GPIC, Aga2–OmcB-*C.tr.* L1, Aga2–OmcB-*C.tr*. E, Aga2–OmcB-*C.tr*. L1 P_66_L or Aga2–OmcB-*C.tr*. E L_66_P were pre-incubated with 500 μg ml^−1^ soluble heparin prior to the adhesion assay. (*n* = 1000 HEp-2 cells, No. experiments = 4, *P* < 0.0001). w/o, without; *C.pn*., *C. pneumoniae*; *C.ca.*, *C. caviae*; *C.tr.*, *C. trachomatis*.

We thus asked if the differences in GAG dependence of infection were caused by sequence alterations found in the various OmcB proteins. OmcB from *C. caviae* GPIC, which shares 82% sequence identity with *C. pneumoniae* OmcB, showed adhesion properties similar to those of the *C. pneumoniae* protein (Fig. 7). The amino acid sequences of *C. pneumoniae* and *C. trachomatis* serovars LGV and E OmcB are 70% identical and all contain the first heparin-binding motif found in *C. pneumoniae* OmcB (Fig. S2). The OmcB proteins from the two *C. trachomatis* serovars L1 and E are very similar (98% identity), differing in only 11 of 547 positions. One of the differences is found in the vicinity of the conserved heparin-binding motif, at residue 66 (proline in *C. trachomatis* serovar L1 and leucine in *C. trachomatis* serovar E). Interestingly, the gor iv secondary structure prediction program indicated that the different amino acids found at position 66 might result in a change from a helical structure (OmcB *C. trachomatis* serovar E) to a coiled-coil structure (OmcB *C. trachomatis* serovar L1). We thus decided to test the effect of changing the proline at position 66 of *C. trachomatis* serovar L1 OmcB to leucine. Analysis of the corresponding OmcB-L1 variant, OmcB-L1 P_66_L, using the yeast adhesion assay, revealed that adhesion was no longer dependent on heparin (Fig. 7). In the converse experiment we changed the leucine at position 66 of *C. trachomatis* E OmcB to proline. The subsequent analysis revealed that the OmcB-E L_66_P variant behaved like the OmcB-L1 protein: addition of heparin led to a 60% decrease in attachment of yeast cells displaying this variant to HEp-2 cells. Thus, our findings strongly imply that heparin dependence of infection can be conferred or reversed by a single-amino-acid alteration in OmcB.

## Discussion

Attachment of a pathogen to its host cell is the crucial first step in the infection process. Pathogenic microorganisms express specific cell surface adhesins which interact with eukaryotic cell surface molecules in order to enable subsequent infection. It has been well documented that GAG molecules play a role in the initial phase of the infection process by chlamydia (Campbell and Kuo, 2006). We have reported previously that one of the earliest steps in *C. pneumoniae* infection is the adhesion of infectious EBs to heparan sulphate-like GAGs on the target cell surface (Wuppermann *et al.*, 2001). The findings presented here imply that the *C. pneumoniae* OmcB protein is the bacterial adhesin that mediates binding to heparan sulphate-like GAGs on human epithelial and endothelial cells, and is important for the establishment of the chlamydial infection. Interestingly, the OmcB proteins from *C. trachomatis* L1 and E, as well as from *C. Caviae*, all share adhesive properties, although *C. trachomatis* L1 and E differ in their dependency on GAGs.

### The yeast display system allows identification and characterization of bacterial adhesin–receptor interactions

In this study we demonstrate for the first time the suitability of the yeast display system for the identification of bacterial adhesins and the dissection of their interactions with host cell receptors. The well-characterized invasin protein (Inv) from *Y. pseudotuberculosis*, when presented on the yeast cell surface, mediated adhesion to epithelial HEp-2 cells in a dose-dependent manner. The observed lateral binding of Inv yeast cells to HEp-2 cells is reminiscent of the zipper-like interaction mechanism of invasin-expressing *Yersinia* cells with human cells, which is thought to maximize adhesin–receptor interactions (Isberg *et al.*, 1987; Dersch and Isberg, 1999). Both invasin-expressing bacteria and latex beads coated with invasin are taken up by HEp-2 cells in a concentration-dependent manner (Dersch and Isberg, 1999). We did not observe uptake of Inv-expressing yeast cells by HEp-2 cells when the adhesion experiment was performed at 37°C (data not shown), suggesting either that the affinity of Inv yeast cells for HEp-2 cells might be insufficient to promote uptake or that the larger size of the yeast cells impairs this process.

Chlamydia most likely uses multiple adhesins on the EB surface for interaction with the host cell (Campbell and Kuo, 2006). We therefore tested other *C. pneumoniae* cell surface proteins and found significant adhesion of GroEL-1-presenting yeast cells to HEp-2 cells (data not shown) (Bavoil *et al.*, 1990; Raulston *et al.*, 1998) (F. Wuppermann, K. Mölleken, C.A. Jantos and J.H. Hegemann, unpublished). In contrast, we observed no adhesion of OmpA-presenting yeast cells to human cells (data not shown). This may indicate that the *C. pneumoniae* OmpA is functionally distinct from the OmpA expressed by the *C. trachomatis* mouse pneumonitis serovar, which has been shown to bind cellular heparan sulphate when incubated as recombinant protein with human cells (Su *et al.*, 1996).

### OmcB is located on the surface of EBs and RBs

In order to act as a cytoadhesin the OmcB protein must be exposed on the cell surface. In our experiments viable *C. pneumoniae* EBs could be stained with the polyclonal anti-OmcB antibody, as was the replicating chlamydial population within the inclusion during the entire infection (Fig. 5A shows infected cells 48 h post infection), indicating that OmcB (or at least part of it) is surface-localized on EBs and RBs during the entire developmental cycle. Recently other groups have also localized the *C. pneumoniae* OmcB on the bacterial cell surface by immune fluorescence microscopy or FACS analysis using polyclonal antibodies raised against the recombinant protein (Montigiani *et al.*, 2002; Vandahl *et al.*, 2002). However, there have been conflicting reports concerning the localization of OmcB in *C. trachomatis* L2 and in *C. psittaci* (Everett and Hatch, 1995; Ting *et al.*, 1995; Mygind *et al.*, 1998; Stephens *et al.*, 2001). Interestingly, in those cases where a surface localization was found, the authors described the specific accessibility of the OmcB N-terminus on the EB cell surface (Ting *et al.*, 1995; Stephens *et al.*, 2001). The surface accessibility of the *C. trachomatis* OmcB N-terminus has also been predicted based on the hydrophilicity profile of the protein (Mygind *et al.*, 1998). These data are in accordance with our finding that the OmcB adhesin function is located within the first 100 N-terminal amino acids.

### The N-terminal region of OmcB is essential for host cell adhesion and infection

The *C. pneumoniae* OmcB protein is very basic: 60 of its 556 amino acids (11%) are arginine or lysine residues. However, the N-terminal region includes a particularly basic domain (aa 41 to aa 79) with 26% basic residues (10 aa/38 aa) and our deletion analysis revealed that the heparan sulphate binding region was localized to this basic domain. Furthermore, yeast cells presenting just the N-terminal 100 amino acids (OmcB_1−100_) including the basic domain exhibited the same adhesion properties as the full-length OmcB protein, indicating that the N-terminal portion is responsible for the entire adhesive activity of OmcB. A detailed amino acid sequence analysis of the basic domain revealed that the OmcB protein carries a sequence motif which perfectly fits the consensus sequence XBBXBX identified previously as one of three major binding motifs found in a variety of heparin-binding proteins, including OmcB (Cardin and Weintraub, 1989; Hileman *et al.*, 1998; Stephens *et al.*, 2001; Capila and Linhardt, 2002). Interestingly, only in the *C. pneumoniae* OmcB protein is this motif duplicated (Fig. 3B). Our mutational analysis of the N-terminal motif (motif I) within the OmcB protein revealed that changing all three basic residues to alanine completely abolished the adhesion of OmcB yeast cells to HEp-2 cells, proving the relevance of the motif for binding of heparan sulphate-like GAGs. It remains to be seen whether motif II present in the *C. pneumoniae* OmcB also contributes to GAG and HEp-2 cell binding. It is possible that the duplication of the heparin-binding motif confers unique properties on the *C. pneumoniae* OmcB protein.

### The OmcB proteins from *C. trachomatis* serovars L1 and E differ in the heparin dependence of their adhesion behaviour

The OmcB protein is highly conserved among chlamydial species (70–84% overall identity). Interestingly, the highest sequence identity of 90–100% is limited to the C-terminal part of the proteins (aa 96 to aa 556), while the basic domain carrying the heparin-binding motif (aa 41 to aa 79) exhibits high sequence diversity, with identity scores of 18–29% only (Mygind *et al.*, 1998). Nevertheless, our analysis revealed that the OmcB proteins from *C. trachomatis* L1 and E and from *C. caviae* all adhere to epithelial human cells with similar affinities. Likewise, it has been shown recently that *E. coli* cells expressing OmcB from *C. trachomatis* L1 adhered to HeLa and Hec-1B cells, and that recombinant OmcB protein, as well as an anti-OmcB antibody, reduced the incidence of *C. trachomatis* infection (Fadel and Eley, 2007).

We assume that host cell binding by these various OmcB proteins always occurs via the N-terminal basic domain carrying the heparin-binding motif. Because the OmcB protein sequences are almost identical among the various *C. trachomatis* serovars, they should have very similar, if not identical, adhesion properties. Surprisingly however, binding of OmcB from serovar E to human cells was found not to be heparin dependent, while OmcB from serovar L1 showed heparin dependency. Even a hundred-fold increase in the amount of heparin, usually sufficient to abrogate any OmcB binding to HEp-2 cells, failed to reduce binding (data not shown). Sequence comparison revealed that within the basic domains only three amino acids differ between the two proteins. The mutational analysis of position 66 in the OmcB proteins from *C. trachomatis* L1 and E identified this position as being crucial for binding of heparin-like GAG structures. We found that the ability of *C. trachomatis* L1 OmcB to bind heparin-like GAGs is completely abrogated by changing the proline residue at position 66 to a lysine. Similarly, adhesion of *C. trachomatis* E OmcB to HEp-2 cells, which is normally completely independent of heparin-like GAGs, can be strongly reduced by prior incubation of the *C. trachomatis* E L_66_P variant with heparin. These findings support and extend the results of other studies which have shown that *C. trachomatis* LGV infections can be blocked by addition of heparin, while infection by serovar E is less susceptible to interference by heparan sulphate or other GAGs tested (Davis and Wyrick, 1997; Taraktchoglou *et al.*, 2001; Beswick *et al.*, 2003). It has been reported that attachment of EBs from *C. trachomatis* trachoma biovar strains B and C to HeLa 229 cells is also only partially inhibited by exogenous heparin (Chen and Stephens, 1997). The OmcB protein sequences of the three trachoma serovars (serovars B, C, E) are identical in the N-terminal basic domain except for the leucine at position 66 which is only found in serovar E. Thus the *C. trachomatis* E L66P variant generated in this work mimics the partial heparin resistance of serovars B and C. The question remains why serovar E is the only trachoma biovar identified thus far, which has evolved the particular OmcB protein exhibiting adhesion to human epithelial cells independent of heparin-like GAGs. The OmcB protein sequences from other chlamydia differ significantly in the N-terminal basic domain from the trachoma biovars, and it is therefore difficult to assess the impact of this particular alteration at position 66 based on sequence comparisons alone. Functional differences in heparin-binding proteins between related species are also observed in other organisms. The *Mycobacterium tuberculosis* adhesin heparin-binding haemagglutinin (HBHA) binds via its C-terminal lysine-rich domain to heparin and is relevant for binding to non-professional phagocytes. The corresponding protein from *Mycobacterium smegmatis* shares 68% identity with its homologue and is associated with the cell wall, but exhibits low affinity for heparin, probably due to a stretch of acidic residues preceding the basic domain (Biet *et al.*, 2007).

Why do the OmcB proteins from *C. trachomatis* serovars L1 and E exhibit different GAG dependencies? Serovar E is thought to be a non-disseminating luminal pathogen, entering and exiting the apical surfaces of columnar epithelial cells lining the mucosa, and ascending via canaliculi in the female genital tract. In contrast, serovar L1 is a disseminating pathogen, exiting the basal surfaces of epithelial cells and then spreading through the submucosa to lymph nodes (Davis and Wyrick, 1997; Davis *et al.*, 2002). Despite these large differences in disease pattern, the genomes of the different *C. trachomatis* serovars share more than 99% identity (Dean *et al.*, 2006), suggesting that small changes in individual genes may have profound effects on the type of disease caused. Our findings are compatible with the idea that the two *C. trachomatis* serovars have evolved specific OmcB adhesins for different niches. It has been demonstrated that heparan sulphate is only localized to the basal membrane of genital columnar epithelial cells of mouse tissue (Hayashi *et al.*, 1987; Bernfield *et al.*, 1999). Thus, *C. trachomatis* serovar E will only be able to adhere to and invade the apical cell side by exploiting a different, non-heparin-like structure, and this might occur via a modified OmcB basic region. It has been suggested that in the *C. trachomatis* strains the *omcB* gene has diverged more rapidly than the *ompA* gene (Millman *et al.*, 2001). This might suggest that selection pressure acted to diversify the N-terminal region of the OmcB protein in order to optimize infection of specific cell types. In contrast, the rest of the OmcB protein, which is probably responsible for the rigid cell wall structure of the EBs and for stabilization of the RB outer membrane during the replicative phase, might face strong stabilizing selection pressure and thus undergoes little change.

### The role of OmcB in chlamydial infection

Glycosaminoglycans have been implicated in cell attachment and invasion by chlamydia species. Here we show that OmcB from *C. pneumoniae* binds to heparin-like GAG structures on human cells and probably is necessary for chlamydial infections. However, the precise chemical nature of the heparin-like structure recognized by OmcB remains to be analysed. Originally we had shown that *C. pneumoniae* strain GiD adheres to heparin-like GAGs on the HEp-2 cell surface (Wuppermann *et al.*, 2001). New data indicate that other *C. pneumoniae* strains and epithelial cell lines as well as endothelial cells exhibit differences in GAG dependency, and evidence for a role of GAGs located on the host cell surface and on the surface of the infectious EB has been presented (Beswick *et al.*, 2003; Yan *et al.*, 2006). Moreover, the infectivity of *C. pneumoniae* strains could also be inhibited by chondroitin sulphate, although heparin treatment was more effective. Taken together, these data suggest that the GAG-binding proteins on the cell surface of different *C. pneumoniae* strains might be variable (Beswick *et al.*, 2003; Yan *et al.*, 2006). Alternatively, other EB surface proteins may modulate the OmcB specificity for certain GAGs or additional unknown GAG-binding proteins may be present on the EB surface.

We propose that the attachment of *C. pneumoniae* to human cells via OmcB–GAG interactions is only the first step towards successful internalization. Blocking this initial interaction between the EB and the target cell by the addition of excess amounts of soluble heparan sulphate (Wuppermann *et al.*, 2001) or recombinant OmcB or OmcB antibody (this work) always results in a residual infectivity of about 10%. This points to the presence of additional, as yet unidentified adhesin–receptor interactions which, in combination with the OmcB–GAG interaction, might account in part for tissue tropism and the spread of the pathogen.

The simplest infection model would postulate that OmcB molecules present on the surface of *C. pneumoniae* EBs bind GAG structures located on the host cell surface in order to initiate attachment. However, at present it cannot be excluded that this bimolecular interaction is accompanied by a trimolecular interaction, in which OmcB located on the EB surface binds specific soluble (pathogen- or host cell-derived) GAGs or GAG-like structures, which in turn are recognized by cellular receptors. This latter mechanism has been proposed for *C. trachomatis* by Stephens *et al.* who have shown that the serovars L2 and D seem not to bind directly to heparan sulphate on the host cell surface (Zhang and Stephens, 1992; Stephens *et al.*, 2006). Such a trimolecular model has also been proposed for a number of other bacteria, which may utilize specific GAGs as molecular bridges to interact with a diverse array of mammalian heparin-binding proteins (MHBPs) in order to optimize pathogen–host interactions (Duensing *et al.*, 1999).

## Experimental procedures

### Bacterial strains, yeast strains and cell culture

*Escherichia coli* strain XL-1 blue (Stratagene) was used for protein expression and plasmid amplification. *C. pneumoniae* GiD, *C. trachomatis* serovars LGV (L1/440/Bu; ATCC No. VR-902B) and E (DK-20; Institute of Ophthalmology, London) were propagated in HEp-2 cells (ATCC No. CCL-23) as described (Roblin *et al.*, 1992; Jantos *et al.*, 1997). Chlamydial EBs were purified using a 30% gastrographin (Schering).

The CHO cell lines CHO-K1 (ATCC CCL-61), CHO pgsD-677 (ATCC CRL-2244) and CHO pgsA-745 (ATCC CRL-2242) were cultured in Ham's F12-K nutrient mixture medium (Invitrogen) (Esko *et al.*, 1985; Lidholt *et al.*, 1992). Human umbilical vein endothelial cells (HUVEC), kindly provided by Axel Gödecke, were cultivated through two passages in endothelial cell medium.

The *S. cerevisiae* strain EBY100 (*MAT***a***ura*3-52 *trp*1 *leu2*Δ1 *his3*Δ200 *pep*4:*HIS3 prb*1Δ1.6R *can*1 *GAL*) used for adhesion experiments was grown in synthetic medium plus 2% raffinose (SR) or 2% galactose (SG) (Sherman, 1991) (Invitrogen) (Boder and Wittrup, 1997).

### DNA manipulations and plasmid construction

Plasmids containing chlamydial genes were generated by homologous recombination in *S. cerevisiae*.

The full-length *omcB* coding sequence or *omcB* variants were amplified by PCR from genomic *C. pneumoniae* GiD, *C. trachomatis* serovar LGV L1/440/Bu, *C. trachomatis* serovar E DK-20 or *C. caviae* GPIC DNA, and either cloned directly into the EcoRI+NotI-digested pYD1 vector (Invitrogen) or cloned into the BglII and EcoRI sites of the pAC-2 vector after attachment of a 5′ sequence encoding a His_6_-Tag. The adhesion domain sequence of *Y. pseudotuberculosis* invasin (aa 790–986) was subcloned from pRI284 (Dersch and Isberg, 1999) into EcoRI+NotI-digested pYD1. Site-specific mutagenesis was performed using the Quick Change II mutagenesis kit (Stratagene). All constructs were sequenced prior to further use.

### Protein expression and affinity purification of his_6_-tagged proteins

For expression of Aga2p or Aga2p fusion proteins in yeast strain EBY100 cells were grown according to the users' manual (Invitrogen) (Boder and Wittrup, 1997).

The expression and purification of His_6_–OmcB fusion proteins was performed under denaturing conditions following the procedure recommended by Qiagen. Proteins were renatured by dialysis against PBS, fractionated by SDS-PAGE and detected by Western analysis with an anti-His antibody (Qiagen).

### Immunoblot analysis

SDS-PAGE and immunoblot analysis were performed as described (Sambrook *et al.*, 1989). For detection of Aga2p fusion proteins, extracts from yeast cells were incubated with 1 U of α-mannosidase for 2 h at 37°C to remove *O*-glycosylation of Aga2 protein, resolved by SDS-PAGE and visualized after incubation with anti-His (Qiagen) and AP-conjugated anti-mouse antibodies (Promega).

### Immunofluorescence microscopy

HEp-2 cells on glass coverslips (12 mm diameter) were washed once with PBS at 48 h post infection, fixed with 3.7% formaldehyde for 1 h and permeabilized with PBS containing 0.5% Triton X-100 or fixed with 96% methanol for 10 min. The monolayer was then washed three times with PBS (Birkelund *et al.*, 1990). For detection of chlamydial inclusions or non-fixed *C. pneumoniae* EBs, either anti-DnaK (Birkelund *et al.*, 1990), anti-OmpA (Dako) or anti-OmcB serum (pre-absorbed, diluted 1:250) was used in combination with a Cy3-conjugated anti-rabbit antibody (Sigma), or a monoclonal FITC-conjugated antibody raised against chlamydial LPS was used (Bio-Rad).

Immunofluorescence microscopy of yeast cells expressing Aga2–Inv or Aga2–OmcB was performed on samples of 5 × 10^6^ cells grown for 24 h under inducing conditions. Cells were washed twice with PBS and fixed on glass slides coated with poly-l-lysine. Slides were then incubated with PBS containing 1% BSA, followed by incubation with anti-invasin (Dersch and Isberg, 1999) or anti-OmcB and Cy3-conjugated anti-rabbit antibodies (Invitrogen, Sigma). Cells were viewed using a Zeiss Axioskop.

### Adhesion assays

Yeast adhesion assay was performed with HEp-2 and CHO cell lines cultivated on glass coverslips. Yeast cells (1 × 10^6^ in 1 ml of PBS) expressing a specific protein on the cell surface were added to 1 × 10^5^ HEp-2 or CHO cells. Coverslips were incubated for 1 h at 4°C with gentle shaking, washed three times with 0.5 ml of PBS and fixed with 3.7% formaldehyde. The number of yeast cells attached to 1000 HEp-2 cells was determined by microscopy. Each experiment was repeated four times. We varied the ratio of yeast to HEp-2 cells, using 1:1, 5:1, 10:1 or 25:1, and found that a ratio of 10 yeast cells per HEp-2 cell gave the most reproducible results. When ratios greater than 25:1 were used it was difficult to determine specific adhesion. For proteolytic digestion of surface-exposed proteins yeast cells (1 × 10^6^) were incubated with proteinase K (4 mg ml^−1^) or trypsin (2.5 mg ml^−1^) for 1 h at 37°C, blocked with 10 mg ml^−1^ BSA for 1 h at room temperature, and washed three times with PBS before addition to the HEp-2 cells. GAG-dependent adhesion was analysed by treatment of HEp-2 or yeast cells with or without 50 U of heparinase I (Sigma) for 1 h at 37°C or by addition of 500 μg ml^−1^ soluble GAGs (heparin, C-6-S, dermatan sulphate or C-4-S; Sigma) to the yeast cells for 1 h at 4°C. Yeast cells were washed three times with PBS prior to the adhesion assays.

Adhesion assays with protein-coated latex beads were performed as described (Dersch and Isberg, 1999). The coating efficiency was analysed by immunoblotting. Latex beads attached to samples of 1000 HEp-2 cells were counted under the microscope in four independent experiments.

### Infection inhibition assays

HEp-2 cells were cultivated on glass coverslips for 48 h to give a confluent monolayer, and incubated with 500 μl of medium containing recombinant protein (20–200 μg ml^−1^ in PBS) for 1 h at 37°C. Purified chlamydial EBs [multiplicity of infection (moi) 100] were added to the protein suspension and incubated for 1 h at 37°C without centrifugation. The high moi was used to facilitate infection in order to avoid the influence of the centrifugation procedure on the adhesion/infection process. After removal of the supernatant, cells were washed three times with PBS and incubated for 48 h in medium containing 1.2 μg ml^−1^ cycloheximide before fixation with methanol. For neutralization studies with anti-OmcB serum, purified chlamydial particles (moi 100) were added to 500 μl of antibody suspension (pre-absorbed, diluted 1:250 in PBS) and incubated for 1 h at 4°C. The mixture was then added to untreated HEp-2 cells and incubated for 1 h at 37°C without centrifugation. After three washes with PBS, cells were incubated for 48 h in medium with cycloheximide before fixation with methanol. Inclusion formation was analysed by immunofluorescence microscopy as described above. The number of inclusions in 80 microscopic fields (20 per coverslip) was determined, and the number found in PBS-treated samples was set to 100%.

### Bacterial adhesion assay

After pre-incubation of HEp-2 cells with 200 μg ml^−1^ recombinant protein or 500 μg ml^−1^ heparin, purified chlamydial EBs (moi 100) were added and incubated for 1 h. Cells were fixed with formaldehyde and adhering EBs were detected with a *C. pneumoniae*-specific primary antibody followed by a horseradish peroxidase (HRP)-coupled secondary antibody (Dako).

### Bacterial adhesion assay of CFSE-labelled *C. pneumoniae* EBs by flow cytometry

Purified *C. pneumoniae* EBs (2 × 10^8^) were labelled for 1 h at 37°C with 25 μmol of CFSE (Molecular Probes) and washed twice with PBS containing 1% BSA as previously described (Schnitger *et al.*, 2007). Confluent monolayers of HEp-2 cells or HUVE cells in 24-well plates were incubated for 4 h at 37°C with 200 μg ml^−1^ recombinant protein or 500 μg ml^−1^ heparin, and then CFSE-labelled *C. pneumoniae* EBs were added (moi 10) for 1 h. Cells were washed with PBS, trypsinized and fixed with formaldehyde, and adhesion was measured by flow cytometry using a FACSAria (BD Biosciences).

### Statistical and bioinformatical analysis

clustal w and gor iv secondary structure prediction tools were used for sequence alignments and secondary structure prediction (http://www.expasy.org/). Statistical analysis of adhesion assays with latex beads, the infection inhibition assay and bacterial adhesion assay was carried out by two-way anova. Statistics for yeast adhesion assays were subjected to a two-sided Wilcox–Mann–Whitney *U*-test and significance was assessed by the Bonferroni method (Krauth, 1988).

## Supplementary material

This material is available as part of the online article from:

http://www.blackwell-synergy.com/doi/abs/10.1111/j.1365-2958.2007.06050.x

(This link will take you to the article abstract).
