# Supplementary material for: The Chlamydia outer membrane protein OmcB is required for adhesion and exhibits biovar-specific differences in glycosaminoglycan binding
Source: Mol Microbiol. 2007 Dec 11;67(2):403–19. doi: 10.1111/j.1365-2958.2007.06050.x (PMC2229832; doi:10.1111/j.1365-2958.2007.06050.x)
Supplement: Supplementary file 1 [file mmi0067-0403-SD1.pdf]

Supplementary Material

The *Chlamydia* outer membrane protein OmcB is required for adhesion and exhibits biovar-specific differences in glycosaminoglycan binding

Moelleken, Katja and Hegemann, Johannes H.
Funktionelle Genomforschung der Mikroorganismen
Heinrich-Heine-Universität Düsseldorf, Germany

Supplementary Figure legends.

Supplementary Figure S1.

Efficiency of adhesion increases with the amount of OmcB present on latex beads.

Top: Western analysis of the OmcB protein present on 1-µm latex beads. After the coating procedure, equal amounts of beads coated with BSA (200 µg/ml) or OmcB protein (12.5, 25, 50 or 100 µg/ml) were prepared for Western blot analysis, separated by SDS-PAGE and probed with an anti-His antibody. The positions of size markers of 98, 64 and 50 kDa are indicated. Bottom: Adhesion of latex beads to HEp-2 cells. Latex beads ($1x10^6$) coated with BSA (200 µg/ml), or with 12.5, 25, 50 or 100 µg of OmcB protein were incubated with $1x10^5$ HEp-2 cells and the numbers of associated beads per 1000 HEp-2 cells was determined by microscopy. (N = 1000 HEp-2 cells, No. experiments = 4, $P < 0.0001$).

Supplementary Figure S2.

Comparison of the heparin-binding motifs and adjacent regions in OmcB proteins from *C. pneumoniae* and the *C. trachomatis* serovars L1 and E.

The heparin-binding motif(s) is (are) underlined. Position 66 in *C. trachomatis* serovars L1 and E is marked with an asterix. The amino acid change at position 66 (proline to leucine in the *C. trachomatis* serovar $L1_{P66L}$; leucine to proline in the *C. trachomatis* serovar E_{L66P}) is indicated by double underlines. Predicted coiled-coil (c) and helical structures (h) are shown below the amino acid sequence.

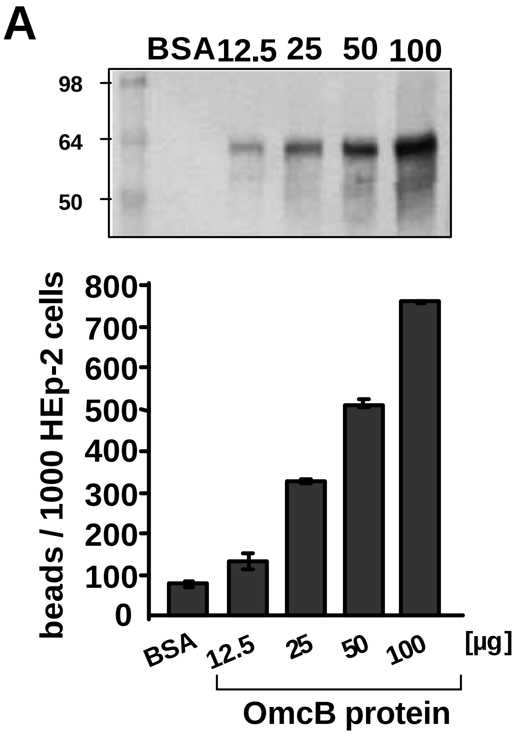

Supplementary Figure S1

```
41                                    79
SAETKPAPVPMTAKKVRL VRRNKQPVEQKSRGAFCDKEF    C. pneumoniae
cccccccccchhhhhhhh hhccccccccccccccccccc

LADTKAKDNTSHKSKKARKNHSKETPVNRKKVAPVHESK     C. trachomatis L1
cccccccccchhhhhhhhhcccccccccccccccccccc

LADTKAKDNTSHKSKKARKNHSKETLVDRKEVAPVHESK     C. trachomatis E
cccccccccchhhhhhhhhccccccchhhhccccccccc

LADTKAKDNTSHKSKKARKNHSKETLVNRKKVAPVHESK     C. trachomatis L1 P66L
cccccccccchhhhhhhhhccchhhhhhhhccccccccc

LADTKAKDNTSHKSKKARKNHSKETPVNRKKVAPVHESK     C. trachomatis E L66P
cccccccccchhhhhhhhhcccccccccccccccccccc
```

CCC — coiled-coiled structure

hhh — helical structure

Supplementary Figure S2
